# Maleic Anhydride-Grafted Isotactic Polybutene-1 and Modified Polyamide 6

**DOI:** 10.3390/polym10080872

**Published:** 2018-08-05

**Authors:** Yongxian Zhao, Chen Ma, Shijie Cheng, Wei Xu, Yuejuan Du, Yansong Bao, Zuojie Xiao

**Affiliations:** Key Laboratory of Rubber-Plastics Ministry of Education/Shandong Provincial Key Laboratory of Rubber-Plastics, Qingdao University of Science and Technology, Qingdao 266042, China; machen_qust@163.com (C.M.); chqust@126.com (S.C.); xuwei1018@163.com (W.X.); duyuejuan0124@163.com (Y.D.); baoyansong1994@163.com (Y.B.); mc13012476689@163.com (Z.X.)

**Keywords:** isotactic polybutene-1, maleic anhydride, divinyl benzene, polyamide 6, modification

## Abstract

Maleic anhydride (MAH)–divinyl benzene (DVB) multi-monomer melt-grafting onto isotactic polybutene-1 (iPB-1) was carried out in a torque rheometer. The effects of dicumyl peroxide (DCP), MAH, and DVB concentrations, and temperature, on the reaction, were investigated. The optimized conditions were 170 °C, DVB/MAH = 4:6 (mass ratio). DVB as a comonomer enhanced the grafting degree (Gd) and grafting efficiency (Ge) of iPB-g-MAH better than styrene. The initiator DCP had little effect on Gd as its concentration over 0.2 phr, but the grafts’ melt flow rate (MFR) increased significantly, and relative molecular weight decreased remarkably with increased DCP concentration. With increasing Gd, the contact angle of grafts with water decreased, and there was a larger crystallization rate. The study of iPB-1 and iPB-g-MAH (Gd = 1.5%)-modified polyamide 6 (PA6) showed that iPB-g-MAH had an obviously toughening effect on PA6. With increasing iPB-g-MAH concentration, the blends of impact strength and elongation at break increased obviously, tensile strength decreased slightly, and MFR decreased prominently, which greatly slowed the processing degradation of PA6. The properties of iPB-1/PA6 blends deteriorated. Both DSC curves and SEM micrographs confirmed that PA6/iPB-g-MAH blends had much better compatibility than PA6/iPB. The reason was that the anhydride group in iPB-g-MAH reacted with amide group in PA6 to improve the compatibility between two phases, and iPB-g-MAH is an excellent modifier for PA6.

## 1. Introduction

The isotactic polybutene-1 (iPB-1), invented by Natta and coworkers [1,2], is a widely used polymorphic polyolefin material [3,4,5] resulting from its excellent physical and mechanical properties, for instance, relatively high heat distortion temperature, low tendency to creep, and high stress-cracking resistance [6,7]. However, the application of iPB-1 was limited due to its disadvantages, such as slow crystallization rate, crystal transformation at room temperature [8,9,10], and low surface activity. At present, its limited application in hot water pipes greatly differs from the wide application of polypropylene (PP) [11,12,13] in the plastic industry. At present, there are many reports about the melt-grafting modification of polyethylene (PE) or PP using maleic anhydride (MAH) as monomer, but few reports have been found regarding the modification of iPB-1. The author’s team has been working on the functional modification of iPB-1 for many years [14,15,16,17,18]; MAH is the most widely used graft monomer, which contains both the double bonds that can be grafted and the functional groups for the subsequent chemical modification and it is not prone to homopolymerization under the processing conditions [19,20]. A previous study [17] showed that the polarity, crystallization rate, and the rate of conversion of form II to form I of iPB-1 were significantly accelerated after grafting MAH. It was also found that the structure and concentration of comonomer had great influence on the structure of iPB-g-MAH. In this study, the effects of new comonomer of divinyl benzene (DVB) on the grafting of MAH onto iPB and the principle of iPB-g-MAH modified polyamide 6 (PA6) were explored, and the purpose of this study is to expand the applications of polybutene-1 into polymer blends and composites.

## 2. Materials and Methods 

### 2.1. Materials

Isotactic polybutene-1 (iPB-1) (identical content 96%) with a melt flow rate of 1.06 g/min at 190 °C and 2.16 kg, in accordance with ISO 1133-1-2011, was supplied by Shandong Dongfang Hongye Chemical Co., Ltd. (Weifang, China). Maleic anhydride (MAH) was from Tianjin Bodi Chemical Co., Ltd. (Tianjin, China). Divinyl benzene (DVB) was supplied by Shanghai Aladdin Biochemical Technology Co., Ltd. (Shanghai, China). Styrene (St) was form Tianjin Guangcheng Chemical Reagent Co., Ltd. (Tianjin, China). Dicumyl peroxides (DCP) was form Shanghai Ebene Chemical Reagents Co., Ltd. (Shanghai, China). PA6 (1013B) was supplied by Ube Japan (Tokyo, Japan). All chemicals were used as received.

### 2.2. Preparation and Purification of iPB-g-MAH

The grafting reaction was carried out in a torque rheometer (XSS-300, Shanghai Kechuang Rubber and Plastics Machinery Co., Ltd., Shanghai, China). A series of formulas of MAH, comonomer (DVB or St), and DCP were dissolved in acetone, and then premixed with iPB-1 (40 g) at room temperature in the beaker. Finally, when all the acetone had volatilized, the mixture was added to the mixing chamber, which was set to the desired temperature and rotor rate (60 rpm). After 8 min, samples were taken out to purify, as reported in the literature [16].

### 2.3. Characterizations

The purified samples were hot pressed into thin films with thickness of 0.1 mm at 160 °C under 10 MPa for 5 min using the 600 T plate press (Huzhou Dongfang Machinery Co., Ltd., Huzhou, China).

The samples were analyzed by FTIR spectroscopy (Vertex70, Bruker, Germany). The appearance of absorption peak at 1780 cm^−1^ (corresponding to the characteristic absorption peak of the carbonyl groups of MAH) and 2720 cm^−1^ (the characteristic absorption peak of iPB-1 skeleton) [21] were integrated, and the ratio of the peak area (Ra_MAH_ = *A*_1780cm^−1^_/*A*_2720cm^−1^_) was the relative grafting degree of MAH. Based on the acid–base titration [16] and infrared results, a calibration curve was constructed to obtain the formula Gd% = 0.3754*A*_1780cm^−1^_/*A*_2720cm^−1^_, which can be used for calculating absolute grafting degree (Gd) of iPB-g-MAH [18].

The samples were tested through MFR (190 °C, 2.16 kg) using GT-7100-MI type equipment (GOTECH, Taiwan), according to ISO 1133-1-2011.

The hot-pressed sample films, left at room temperature for one week, were analyzed by a contact angle measuring instrument (DH-HV1351UM type, Shanghai Zhongchen Digital Technology Equipment Co., Ltd., Shanghai, China), using distilled water as a measuring liquid [22]. 

The isothermal crystallization process of the samples was observed by professional polarizing microscope (OLYMPUS BX5, OLYMPUS, Tokyo, Japan). The sample was heated from room temperature to 160 °C at a rate of 10 °C/min, then, maintained for 10 min, and cooled down to 85 °C at a rate of 60 °C/min. The isothermal crystallization process occurred at 85 °C, and the crystal form was recorded per minute.

The Gd of iPB-g-MAH prepared in a double screw extruder (SHJ-30, Nanjing JieEnte Electromechanical Co., Ltd., Nanjing, China) was 1.5%. iPB, iPB-g-MAH (Gd = 1.5%) and PA6 were mixed in a high-speed mixer and uniformly mixed and extruded for granules. The injection molding machine (F2v130, Donghua Machinery Co., Ltd., Dongguan, China) was used to inject the extrudates into standard samples for performance testing. Tensile strength and elongation at break were measured according to ISO 527-1993. The test specimens were type I. Notched impact strength were tested according to ISO 179-1993. Shore D hardness were tested according to ISO 7619-1986. Vicat softening point were tested according to ISO 306-1994.

The thermal analyses of PA6/iPB-g-MAH and PA6/iPB were carried out with a differential scanning calorimeter (model NETZSCH-204, NETZSCH, Selb, Germany). The sample was heated from room temperature to 260 °C, then cooled down to room temperature, then heated to 260 °C at a rate of 10 °C/min, under constant nitrogen flow. Temperature and heat flow scales were calibrated using a standard indium sample.

Scanning electron microscope (JSM-7500F, Japan Electronics Co., Ltd., Tokyo, Japan) was used to observe the morphology of impact sections of different samples.

## 3. Results

### 3.1. The Grafting Reaction of DVB/MAH onto iPB-1

**Figure 1:**
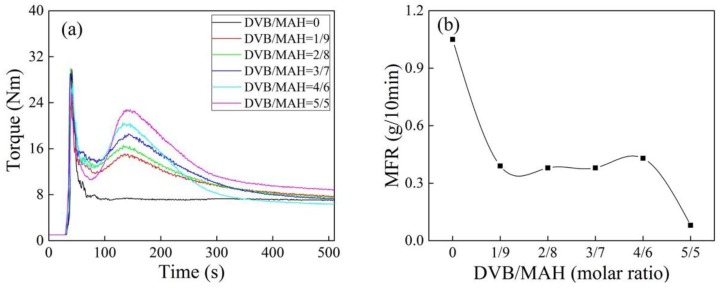
Torque/time spectra (**a**) and MFR (**b**) of isotactic polybutene (iPB)-g-maleic anhydride (MAH) at different divinyl benzene (DVB) concentrations (*T* = 180 °C, dicumyl peroxide(DCP) = 0.6 phr, MAH = 6 phr).

The torque behavior of iPB-g-MAH at different DVB concentrations was examined with a torque rheometer (Figure 1a). The reaction torque peaks of graft products appeared with the addition of DVB, and its intensity gradually strengthened when DVB concentration increased. The reason was that the addition of DVB promoted the reaction of MAH with iPB-1 [23]. The end-torque values increased when DVB concentration increased, reflecting the crosslinking trend [24], this trend was consistent with the decrease of MFR in Figure 1b.

**Figure 2:**
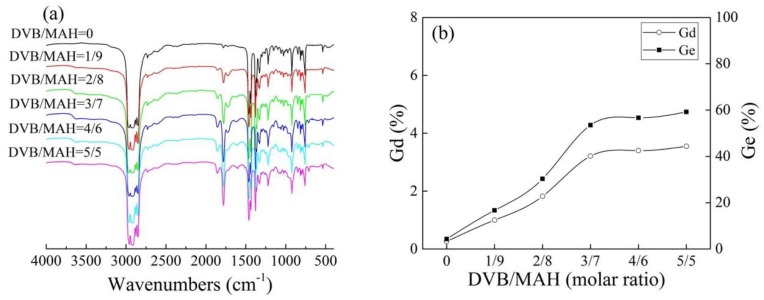
FTIR spectra (**a**) and Gd/Ge (**b**) of grafted products at different DVB concentrations.

Figure 2a shows the FTIR spectra of grafted products at different DVB concentrations. Compared with iPB-g-MAH, the sample of iPB-g-MAH-co-DVB had obvious absorption peaks at 1780 cm^−1^, corresponding to the carbonyl group (C=O) of MAH [25]. This demonstrated that MAH has been grafted onto iPB-1 successfully. Moreover, the absorption peaks of 711 cm^−1^, due to the benzene ring of DVB, indicated that DVB was also grafted onto the macromolecular chains. The peak intensity of grafted products absorption peaks at 1780 cm^−1^ increased when DVB concentrations increased, indicating that DVB significantly promoted the Gd of MAH. The Gd in Figure 2b was calculated by the formula (Gd% = 0.3754*A*_1780cm^−1^_/*A*_2720cm^−1^_) and Figure 2a. It can be seen that the Gd and grafting efficiency (Ge) of the grafted products increased when DVB concentrations increased. The reactivity of DVB/iPB-1 macroradicals was higher than that of MAH/iPB-1 macroradicals. Therefore, DVB and iPB-1 macroradicals first converted to styryl macroradicals, then reacted with MAH to grafted products during the grafting reaction.

**Figure 3:**
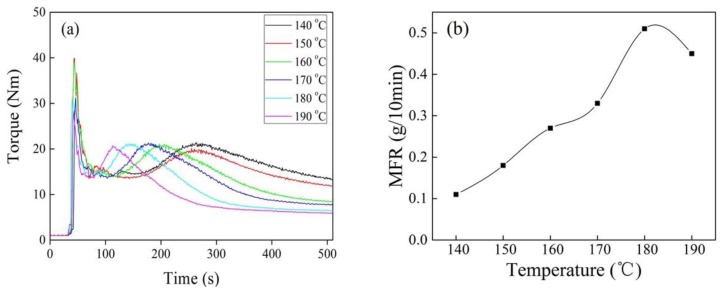
Torque/time spectra (**a**) and MFR (**b**) of iPB-g-MAH at different reaction temperature. (DCP = 0.6 phr, MAH = 6 phr, DVB/MAH = 4:6.)

Figure 3 shows torque and MFR of iPB-g-MAH at different reaction temperature. The reaction torque peak appeared earlier with increasing of temperature, the reason was that the decomposition of DCP increased. The DCP half-life is 9.2 min at 150 °C, and only one min at 170 °C, and the reaction continued due to sufficient free radical concentration. There were two reasons for the reduction of torque: one reason was high temperature which exacerbated the degradation of iPB-1 macromolecules; the other was the viscosity of iPB-1 macromolecules, which decreased when temperature increased. MFR showed in Figure 3b proved that the degradation of iPB-1 macromolecules was the main reason.

**Figure 4:**
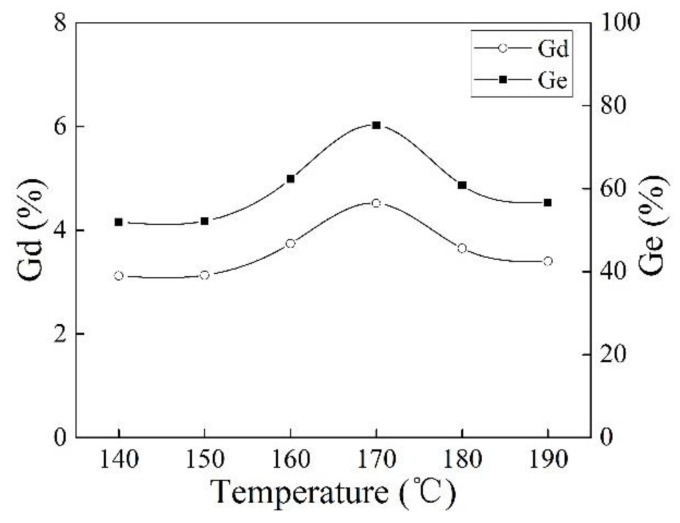
Effect of reaction temperature on Gd and Ge of iPB-g-MAH.

Figure 4 shows the effect of reaction temperature on Gd and Ge of iPB-g-MAH. The Gd and Ge of iPB-g-MAH increased at 170 °C. This can be explained by the fact that the decomposition rate of DCP and the reaction rate were matched, and free radical concentration was appropriate at 170 °C. When the temperature was too high, the decomposition rate of DCP and the transient concentration of free radicals increased rapidly. Meanwhile, the termination rate of free radicals increased, causing the effective concentration of free radicals to decrease, and as a result, the Gd of iPB-g-MAH decreased.

**Figure 5:**
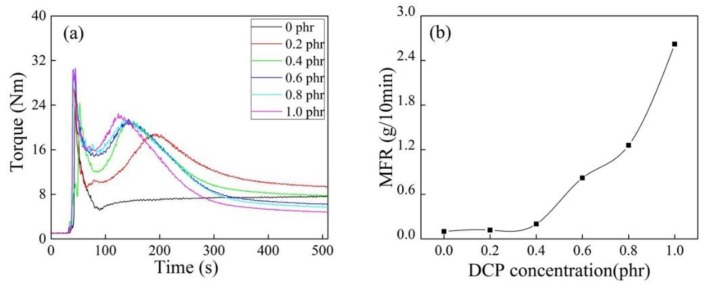
Torque/time spectra (**a**) and MFR (**b**) of iPB-g-MAH at different DCP concentrations. (*T* = 180 °C, MAH = 6 phr, DVB/MAH = 4:6).

It can be seen from Figure 5a that grafting copolymerization had difficulty proceeding without DCP. The reaction torque peak moved forward, and the torque end values decreased with increasing DCP concentration, indicating that the existence of DCP promoted the degradation of iPB-1 macromolecules. This was confirmed by the apparent increase of MFR in Figure 5b.

**Figure 6:**
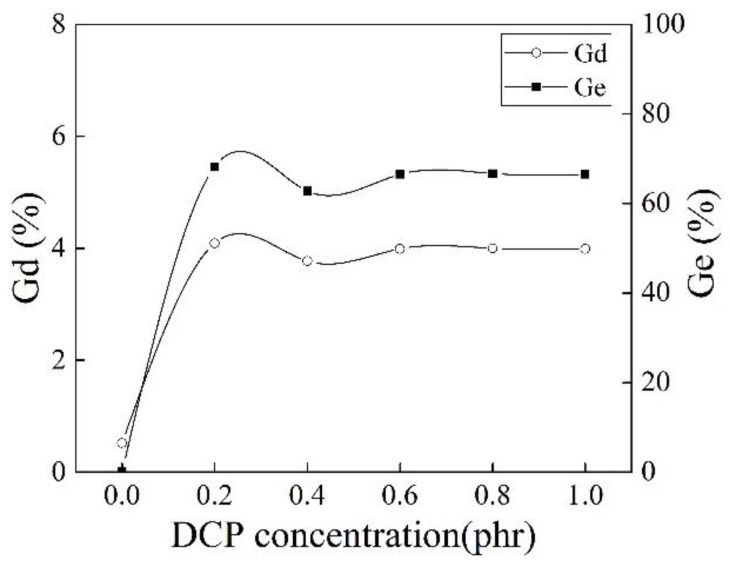
Effect of different DCP concentrations on Gd and Ge of iPB-g-MAH.

It can be seen from Figure 6 that DCP as the initiator significantly promoted the grafting copolymerization of MAH. The initiator DCP had little effect on Gd when its concentration was higher than 0.2 phr, but the MFR of iPB-g-MAH increased significantly (as shown in Figure 5b). It is possible to prepare iPB-g-MAH with the same grafting rate and meeting the different processing requirements in industry.

**Figure 7:**
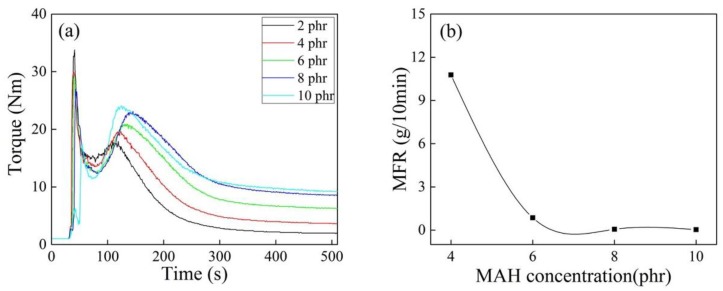
Torque/time spectra (**a**) and MFR (**b**) of iPB-g-MAH at different MAH concentrations. (*T* = 180 °C, DCP = 0.6 phr, DVB/MAH = 4:6).

It can be seen from Figure 7 that the torque end values of iPB-g-MAH increased with increasing MAH concentration, and the degradation of iPB-1 macromolecules caused by DCP was inhibited by the addition of MAH.

**Figure 8:**
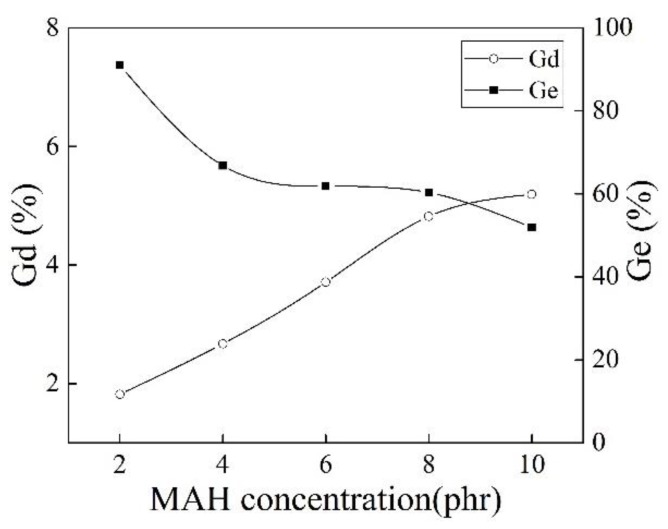
Effect of different MAH concentrations on Gd and Ge of iPB-g-MAH.

Figure 8 shows effect of different MAH concentrations on Gd and Ge of iPB-g-MAH. The increase of Gd of iPB-g-MAH could be attributed to the increase in MAH concentration, owing to an increase in the monomers in the grafting system, as well as the participation in the grafting reaction of more macroradicals. However, the Ge of iPB-g-MAH decreased, and the reason was that the proportion of MAH that participated in the reaction decreased.

It can be seen from Table 1 that the effect of DVB as a comonomer enhanced the Gd and Ge of iPB-g-MAH better than St. Table 2 shows the *Q*–*e* values of different monomers. DVB and St both have negative e values so that both of them can provide one electron to MAH that changed the symmetry of MAH double bond and increased the reactivity of MAH and iPB-1 macroradicals.

According to the Alfrey–Price Q-e deformation formula [15], the reactivity ratio can be calculated and used to compare the difficulty degree of reaction between comonomers and MAH. The reactivity ratio is calculated, that *r*_St_ = 0.379, *r*_DVB_ = 0.012. As a result of 0 < *r*_DVB_ < *r*_St_ < 1, compared with St, DVB copolymerizes with MAH more easily, so that the Gd of DVB/MAH onto iPB-1 is higher than that of St/MAH.

### 3.2. Surface Properties and Crystallization Properties of iPB-1 and Its Grafts

**Figure 9:**
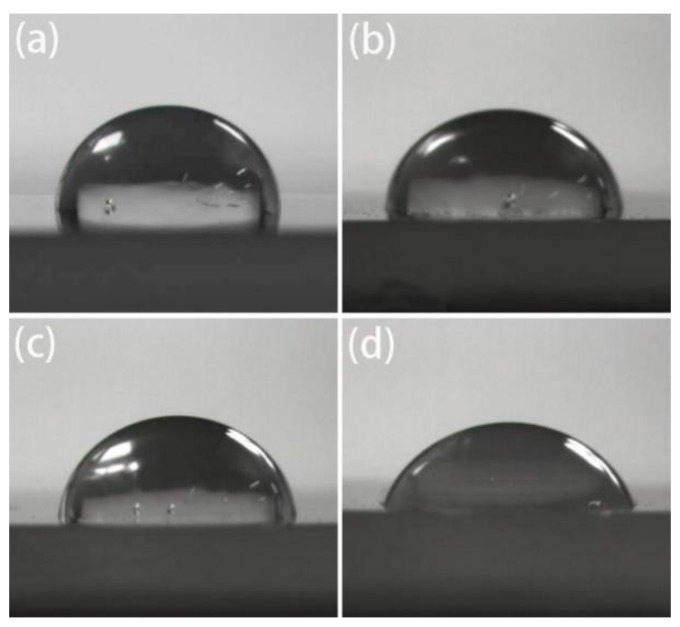
Contact angles of iPB-1 and iPB-g-MAH samples with different Gd: (**a**) iPB-1; (**b**) Gd = 1.09%; (**c**) Gd = 2.74%; (**d**) Gd = 4.1%.

**Figure 10:**
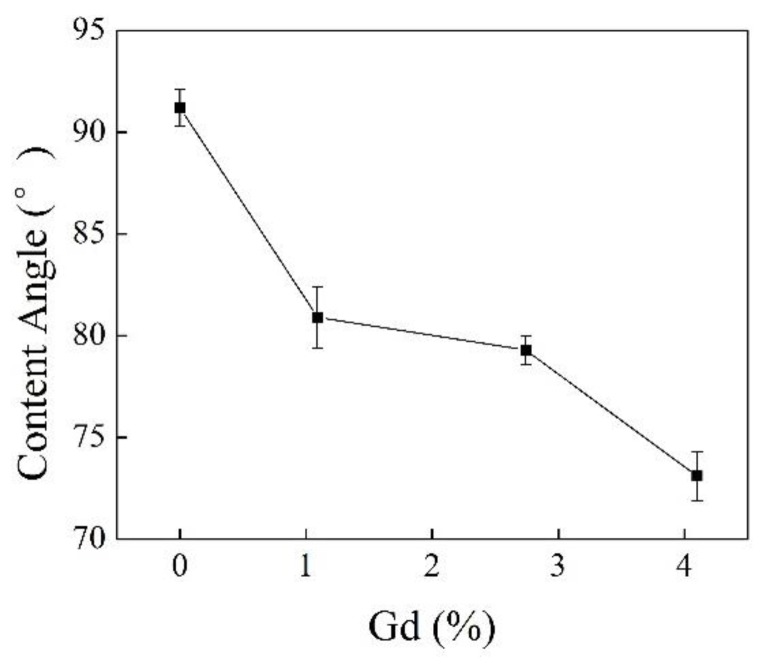
Contact angles of iPB-1 and iPB-g-MAH samples with different Gd.

Figure 9 and Figure 10 shows the contact angle with water decreased with the increase of grafting degree, indicating that surface polarity increased and the graft chains of MAH effectively improved the polarity and hydrophilicity of iPB-1.

**Figure 11:**
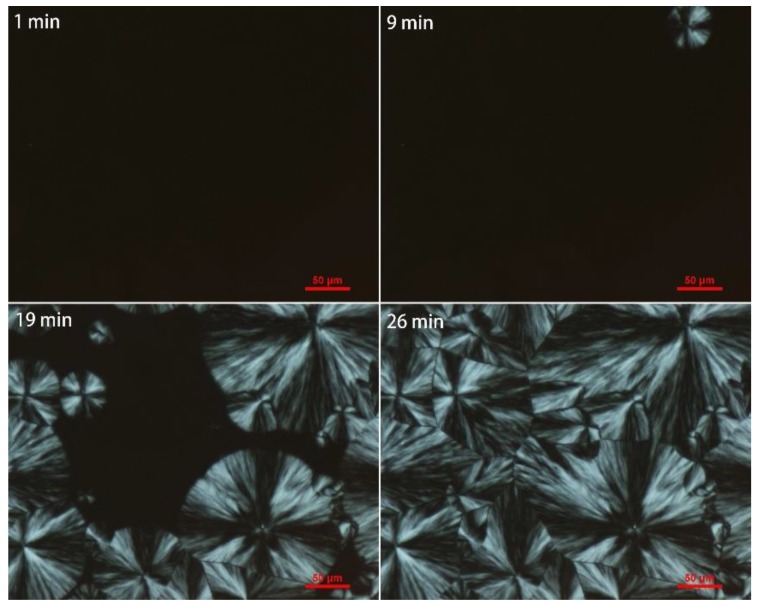
Polarizing optical micrograph of iPB-1 at 85 °C with changing time.

**Figure 12:**
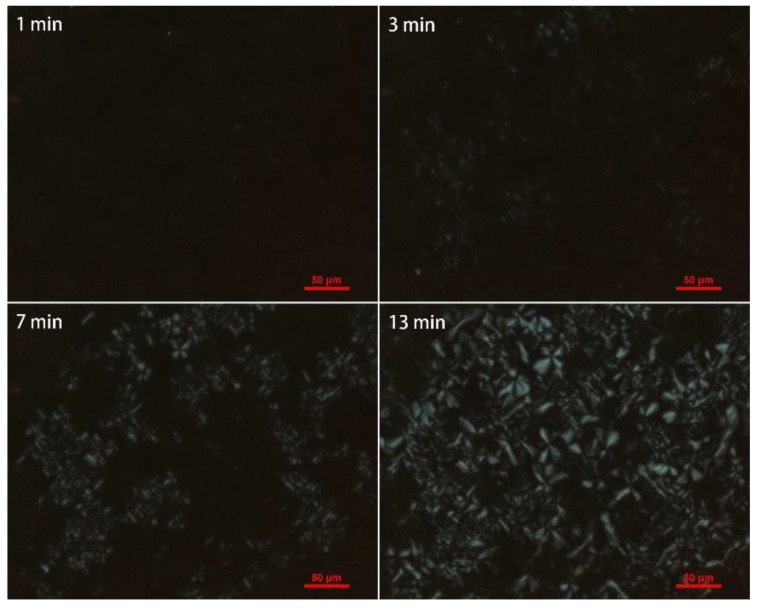
Polarizing optical micrograph of iPB-g-MAH (Gd = 1.563%) at 85 °C with changing time.

As shown in Figure 11 and Figure 12, grafted sample exhibited smaller crystals than iPB-1 at the same amplification factor, and the spherical crystal of iPB-1 took 26 min to complete, while the grafted sample only needed 13 min, indicating that a decrease of the radius of the spherulites, an increase of crystal nuclei density, and crystallization rate is because of polar MAH graft chains.

### 3.3. iPB-g-MAH Modified PA6

The iPB-g-MAH (Gd = 1.5%, MFR = 15.1 g/10 min) or iPB-1 was used to modify the PA6.

**Figure 13:**
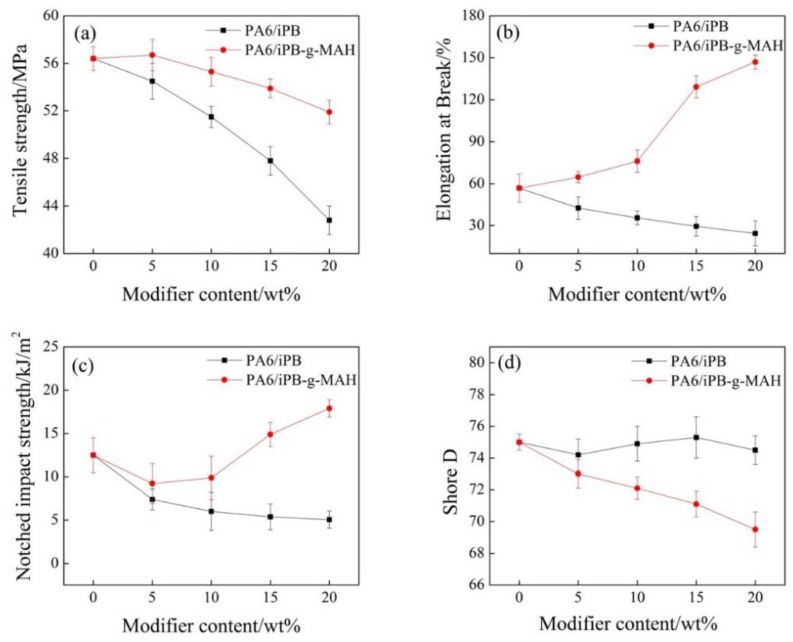
Effect of content of iPB and iPB-g-MAH in blends on blends mechanical properties. (**a**) Tensile strength; (**b**) Elongation at break; (**c**) Notched impact strength; (**d**) Shore D.

As shown in Figure 13, the elongation at break, notch impact strength, and tensile strength of PA6/iPB blends decreased significantly when the iPB-1 concentration increased. It suggested that iPB-1 should not be used to modify PA6. For PA6/iPB-g-MAH blends, the elongation at break and notch impact strength increased when iPB-g-MAH concentration increased, the elongation at break of blends (20% content of iPB-g-MAH) increased from 56.8% to 147%, while the tensile strength only slightly decreased. The result showed that the anhydride group in iPB-g-MAH reacted with the amide group in PA6, and improved the compatibility between iPB-g-MAH and PA6. This can be confirmed by the Figure 16. The iPB-g-MAH is an excellent modifier for PA6. This also explained the fact that iPB-1 had little effect on the hardness and heat resistance temperature of the blends, while iPB-g-MAH had relatively greater influence on them.

**Figure 14:**
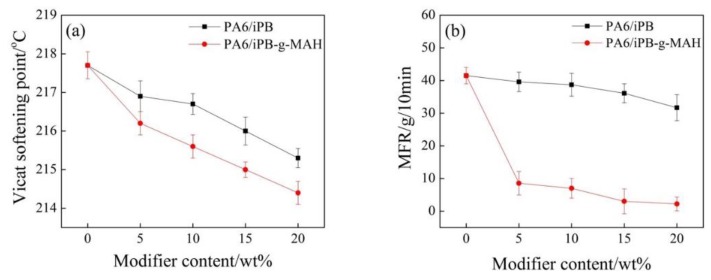
Effect of content of iPB and iPB-g-MAH in blends on Vicat softening point (**a**) and MFR (**b**).

The addition of iPB or iPB-g-MAH did not affect the Vicat softening point temperature of the blends (Figure 14). This can be explained by the fact that the heat resistance of iPB-1 and iPB-g-MAH were significantly lower than that of PA6. The MFR of PA6/iPB-g-MAH blends much lower than that of PA6/iPB, which greatly slowed the degradation of PA6 during processing. The reason was that the anhydride group in iPB-g-MAH reacted with the amide group in PA6 and formed crosslinking structure. This resulted in higher relative molecular weight of PA6 and improved its processability.

**Figure 15:**
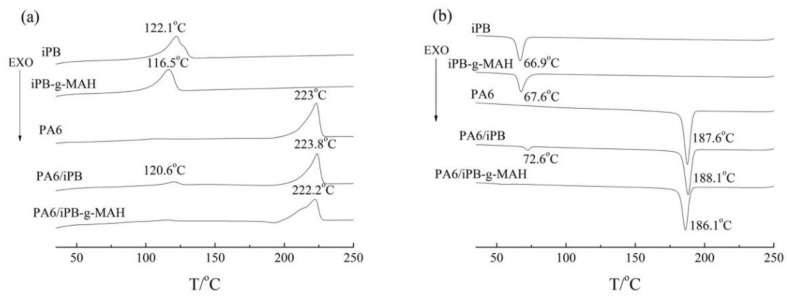
DSC melting curves (**a**) and crystallization curves (**b**) of different samples.

The melting and crystallization curves of iPB-1, PA6, iPB-g-MAH, PA6/iPB-g-MAH (85:15), and PA6/iPB (85:15) are shown in Figure 15. In PA6/iPB blends, PA6 and iPB exhibited independent crystallization peaks because of weak interaction between two phases. The interference between two crystalline phases had a slight effect on the melting and crystallization temperature. Moreover, the crystallization of PA6 was affected by the addition of iPB, resulting in its degree of crystallinity decreasing (Table 3). However, only one melting and one crystallization peak were shown in DSC curves of PA6/iPB-g-MAH blends. The iPB-g-MAH crystallization peak disappeared, and the melting peak and crystallization peak of PA6 moved to low temperature. This indicated good compatibility between PA6 and iPB-g-MAH. The reason may be that the chemical reaction between the acid anhydride group in iPB-g-MAH and the amide group in PA6 formed crosslinking structures. This was confirmed by the apparent decrease of its MFR. It was difficult to crystallize because of crosslinking structure causing the degree of crystallinity of PA6 to decrease (Table 3).

**Figure 16:**
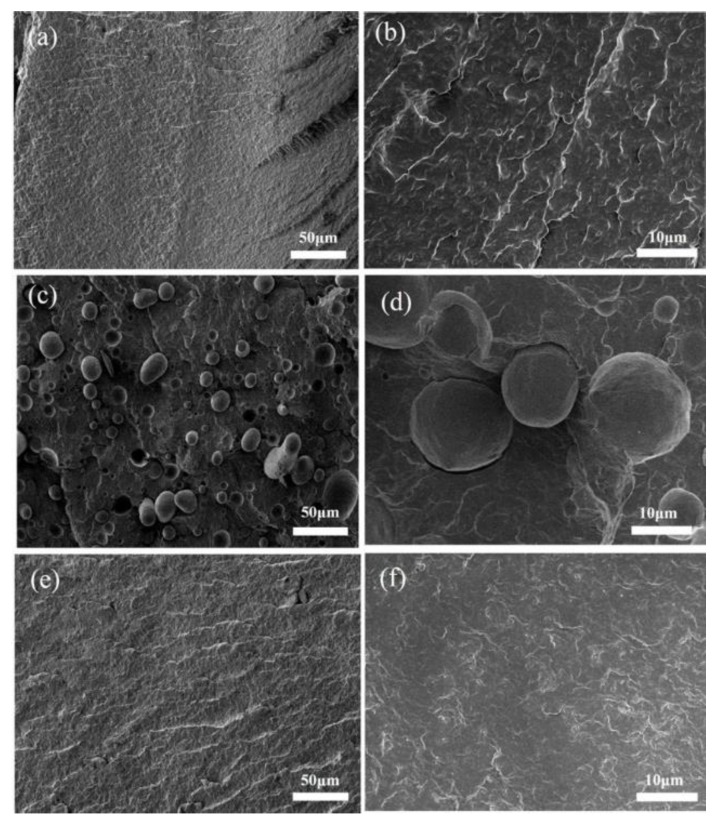
SEM fractographs of different samples: (**a**,**b**) PA6; (**c**,**d**) iPB/PA6 blends; (**e**,**f**) iPB-g-MAH/PA6 blends.

The SEM of PA6/iPB (85:15) and PA6/iPB-g-MAH (85:15) blends are shown in Figure 16. In PA6/iPB blends, the interface was clear between the two phases, due to lack of cohesion. The iPB dispersion phase showed smooth spheroids or ellipsoids that were pulled out and left holes or remained as hemispherical protrusions on the fracture surface (Figure 16c,d). In PA6/iPB-g-MAH blends, the interface between two phases was obscure, and the dispersion phase iPB was virtually invisible, indicating that the interaction between them was enhanced and macroscopic two-phase compatibility was achieved. This also can be confirmed by the Figure 13, Figure 14 and Figure 15.

## 4. Conclusions

The addition of DVB as a comonomer enhanced the Gd and Ge of iPB-g-MAH. The optimized conditions were 170 °C, DVB/MAH = 4:6 (mass ratio). The initiator DCP had little effect on Gd as its concentration over 0.2 phr, but MFR of iPB-g-MAH increased significantly, making it possible to prepare iPB-g-MAH with the same grafting rate and meeting the different processing requirements in industry. The surface polarity of iPB-g-MAH increased with the increase of Gd, and its crystallization rate was higher than iPB.

The study of iPB-g-MAH (Gd = 1.5%) modified PA6 showed that the iPB-g-MAH had an obviously toughening effect on PA6. In PA6/iPB-g-MAH blends, with increasing iPB-g-MAH concentration, its impact strength and elongation at break increased significantly, the tensile strength decreased slightly, and especially the MFR decreased significantly, which greatly reduced the degradation of PA6 during processing. However, the properties of iPB-1/PA6 blends deteriorated. The reason was that the anhydride group in iPB-g-MAH reacted with the amide group in PA6, which improved the compatibility between iPB-g-MAH and PA6. This can be confirmed by the DSC results. In PA6/iPB-g-MAH blends, only one melting and one crystallization peak were observed in DSC curves. The SEM showed that the interface between the two phases of PA6/iPB-g-MAH blend disappeared.

## Figures and Tables

**Table 1:** The Gd and Ge of grafted iPBs with different comonomers.

	Reaction Temperature (°C)	DCP Concentration (phr)	MAH Concentration (phr)	Comonomers Concentration (phr)	Gd (%)	Ge (%)
iPB-g-MAH	180	0.6	6	0	0.26	4.3
iPB-g-MAH-co-DVB	180	0.6	6	4	3.4	56.7
iPB-g-MAH-co-St	180	0.6	6	6	1.62	27

**Table 2:** *Q*–*e* values of MAH and comonomers.

	MAH	St	DVB
Q	0.23	1	3.350
e	2.25	−0.8	−1.770

**Table 3:** DSC data of PA6.

Samples	Degree of Crystallinity
PA6	39.7%
PA6/iPB	33.4%
PA6/iPB-g-MAH	27.8%
